# Integrated Care Delivery for HIV Prevention and Treatment in Adolescent Girls and Young Women in Zambia: Protocol for a Cluster-Randomized Controlled Trial

**DOI:** 10.2196/15314

**Published:** 2019-10-03

**Authors:** Sujha Subramanian, Patrick Edwards, Sarah T Roberts, Maurice Musheke, Michael Mbizvo

**Affiliations:** 1 RTI International Waltam, MA United States; 2 RTI International Research Triangle Park, NC United States; 3 RTI International San Francisco, CA United States; 4 Population Council Lusaka Zambia

**Keywords:** HIV, prevention, treatment, Zambia, adolescent girls and young women

## Abstract

**Background:**

Among countries in sub-Saharan Africa, Zambia has one of the highest incidences of HIV. Adolescent girls and young women (AGYW) are a particularly affected group because of their social and economic vulnerability.

**Objective:**

The goal of this study is to test a multilevel package of interventions at the community and health system levels in Zambia in order to connect AGYW with a source of regular care, which will in turn allow for sustainable, successful implementation of regular HIV testing and adherence to antiretroviral treatment.

**Methods:**

We will adapt prior tools to create the SHIELD (Support for HIV Integrated Education, Linkages to Care, and Destigmatization) intervention to educate and empower Zambian AGYW of 10-24 years of age and their families and to create community-based youth clubs to foster peer support. We will also develop integrated wellness care clinics to offer a youth-friendly environment that provides tailored clinical services. We will perform formative research, including focus groups and in-depth interviews, among AGYW, caregivers, and stakeholders to help inform the development and tailoring of the interventions. A cluster-randomized controlled trial will be implemented in Lusaka, with six clinic catchment areas randomized into three groups: zones with integrated wellness care clinics and SHIELD intervention, zones with only SHIELD intervention, and control zones with no intervention. We will assess HIV testing among the HIV-negative or unknown (HIV-/u) cohort, and retention in care along with viral load suppression will be evaluated in the HIV-positive (HIV+) cohort. We will use in-depth interviews and surveys to collect staff and stakeholder feedback after the trial. Cost-effectiveness of the interventions and return-on-investment impacts will be quantified using a microsimulation model.

**Results:**

Interim results are expected in 2021, and the final results are expected in 2022. If this multilevel intervention is successful in establishing a comprehensive care continuum for HIV-affected AGYW, the Zambian Ministry of Health may advocate for expansion to additional settings to support national scale-up.

**Conclusions:**

This integrated service delivery model can also be a platform to implement additional preventive services, so HIV-/u and HIV+ AGYW can receive comprehensive, integrated services.

**Trial Registration:**

ClinicalTrials.gov NCT03995953; https://clinicaltrials.gov/ct2/show/NCT03995953

**International Registered Report Identifier (IRRID):**

PRR1-10.2196/15314

## Introduction

Zambia is experiencing one of the highest incidences of HIV in the world, and adolescent girls and young women (AGYW) are a particularly affected group because of their social and economic vulnerability [1,2]. Approximately 5% of girls aged 15-19 years and 11% of young women aged 20-24 years are living with HIV in Zambia, with about 14,000 new infections among AGYW annually [1,3,4]. HIV counseling and testing are the key entry point for many HIV prevention interventions and essential for early linkages to HIV treatment [2]. With optimal use of antiretroviral therapy (ART), early diagnosis can reduce transmission to others and improve health outcomes [5-7].

The latest Zambian estimates indicate that only 42% of youth aged 15-24 years know their HIV status, 78% of those diagnosed with HIV are in treatment, and 71% of those in treatment have achieved viral suppression, resulting in a community viral load suppression (at the population level) of less than one-third of the total [8]. Evidence indicates that girls have less-comprehensive HIV knowledge than boys; face gender norms that increase their susceptibility, such as the view that women should never refuse sex with their husbands; and lack access to sexual and reproductive health services to support their reproductive rights [9,10].

The Zambian government prioritizes HIV services for adolescents and encourages evaluation of new models of care delivery to support “test and start” guidelines. Zambia has introduced comprehensive sexuality education, which is meant to be integrated in school curriculums, and is in the early stages of initiating facility-based ART adherence clubs [11]. To spearhead further progress, the government is interested in evaluating new models of HIV testing and treatment. Under the new “test and treat” guidelines [12], linkage to HIV care and retention in care are crucial to the success of ART treatment. A recent systematic review investigating the acceptability of HIV counseling and testing in children and youth aged 5-19 years in sub-Saharan Africa reported that provider-initiated testing and counseling achieved the highest acceptability (86%), followed by home-based HIV counseling and testing (84.9%) and school-linked HIV counseling and testing (60.4%) [13]. There is also some evidence that locating testing and care services within the same facility can improve care linkage and ART initiation [14-16]. Additional evidence is required on whether provider-initiated HIV counseling and testing in an adolescent-friendly, clinic-based setting that is colocated with HIV services can increase HIV testing and improve linkages to HIV treatment among AGYW.

Individual and interpersonal factors such as self-efficacy and social support are key facilitators of HIV testing and ART adherence among AGYW [17]. The transitions from child to teenager to young adult are crucial developmental phases that require age-appropriate interventions [17]. Training tools such as the Stepping Stones program and the Adolescent Girl Empowerment Program (AGEP) are proven to improve self-efficacy and increase HIV knowledge [18-21]. Additionally, during adolescence, both family support and peer influence are important [22,23]. Youth clubs are an important venue to help young people establish friendships and social bonds to support optimal HIV prevention and treatment behaviors [24].

Prior studies and initiatives have attempted to provide youth-friendly services through adolescent ART clinics that offer a range of services, but these clinics have faced challenges because of the stigma associated with HIV and the fear of privacy loss [25-27]. To address this gap, we are developing and testing an integrated wellness care delivery model that targets all HIV-affected AGYW, including those who are HIV-negative or whose status is unknown (HIV-/u) and those who are HIV-positive (HIV+). We will adapt the successful Zambian cervical cancer screening program, which provides a range of services to older women with and without HIV, to offer tailored services to AGYW [28,29]. The availability of the human papillomavirus (HPV) vaccine, which is targeted at AGYW (ages 10-24 years included in this study) and well-accepted in Zambia [30,31], provides an ideal opportunity to address the dual burden of HIV and cervical cancer. The integrated wellness care clinic will offer HIV testing, HIV treatment (in coordination with HIV clinic and dispensary in the same facility), HPV vaccination, and other HIV and sexual and reproductive health care services. Because AGYW experience individual and interpersonal barriers in seeking HIV and sexual and reproductive health services, we will also include a community-based behavioral intervention for AGYW and their families that is specifically tailored to the development stage of the AGYW.

The overall goal of this study is to develop and test a multilevel package of interventions to connect AGYW in Zambia with a source of regular care, which will in turn provide a sustainable, successful implementation of regular HIV testing and support for linkage to care, retention in care, and adherence to antiviral treatment. This approach will avoid siloes and create a comprehensive HIV care continuum with a holistic, integrated health care delivery approach, which is recommended by the Zambian Ministry of Health guidelines for treatment and prevention of HIV [5].

## Methods

### Summary

**Figure 1:**
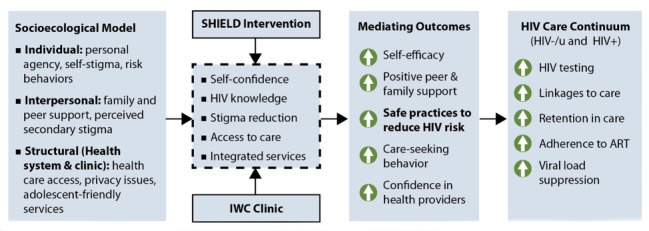
Conceptual framework for creating a comprehensive care continuum for HIV-affected adolescent girls and young women. HIV-/u: HIV-negative or unknown; SHIELD: Support for HIV Integrated Education, Linkages to Care, and Destigmatization; IWC: integrated wellness care; ART: antiretroviral therapy.

To meet the overall goal, we will adapt prior tools to create the SHIELD (Support for HIV Integrated Education, Linkages to care, and Destigmatization) intervention to educate and empower AGYW and their families and to create community-based youth clubs to foster peer support. We will also create the adolescent-friendly integrated wellness care clinics to offer integrated services targeted at AGYW. The theoretical framework, based on the socioecological model, is presented in Figure 1. We will test the hypothesis that the combination of integrated wellness care clinics and age-appropriate SHIELD interventions for AGYW and their families will increase HIV testing, retention in care, and viral load suppression compared to the current standard-of-care clinical services with or without the SHIELD intervention.

### Interventions

#### Support for HIV Integrated Education, Linkages to Care, and Destigmatization Intervention

The SHIELD intervention is based on social cognitive theory, which posits that positive behavior change requires knowledge and skills to increase behavioral capability, self-efficacy to increase the belief that one can achieve the desired outcomes, and social support to provide positive reinforcement and develop positive outcomes expectations [32]. We will develop a modular-based program for AGYW that increases their knowledge, skills, and self-efficacy to seek care. We also will develop an education program for family members to increase social support for AGYW. Intervention content for both AGYW and families will be tailored for five distinct groups to reflect AGYW’s development stage and HIV status. The groups include ages 10-12, 13-15, and 16-20 years for HIV-/u AGYW, and ages 16-20 and 21-24 years for AGYW living with HIV. Modules will be adapted based on formative research (focus group and in-depth interviews ) using existing evidence-based interventions such as Stepping Stones [20,21] and Families Matter! [33,34]. Modules will address HIV prevention and treatment, general wellness and sexual and reproductive health, approaches to combat stigma and discrimination, and skills for better communication and include new content on health service availability and access to increase self-efficacy in seeking health care services. In addition to the education modules, we will establish youth clubs for the five distinct groups by adapting those created for the AGEP initiative and other similar programs [18,19]. Youth clubs will be facilitated by peer navigators, who are AGYW aged 16-24 years and have been trained in participant confidentiality, AGYW rights, basic counseling skills, sexual and reproductive health, HIV continuum of care, processes of referral and linkages for health services, recruitment strategies, and study aims.

#### Integrated Wellness Care Clinic Intervention

Integrated wellness care clinics will be created where AGYW can receive sexual and reproductive health care services, including HIV testing and treatment, family planning, sexually transmitted disease screening and treatment, and HPV vaccination. Integrated wellness care clinics will be established in existing government health facilities and will follow the model of the cervical cancer screening clinics that have been embedded within government facilities to provide comprehensive services to women [28,29]. Standard operating procedures (SOPs) will be developed, covering HIV, HPV vaccination, and sexual and reproductive health clinical guidelines specific to AGYW; care pathways mapped out for common problems or conditions; procedures to maintain patient privacy; quality assurance checklists; and details on documentation and data capture (preprogramed computer tablets will be used to enter study data). We will also map out the physical structure and layout of the room for the integrated wellness care to ensure ergonomics in patient care processes and offer privacy for physical exams and counseling. We will explore the best options for placement of the integrated wellness care clinic within the health facilities with the use of at least two rooms: one devoted to the intake process and another to medical examinations. To accommodate AGYW, who are generally not available to attend clinics during work or school hours, the integrated wellness care operational hours will include early morning, late evening, and weekend hours.

Integrated wellness care clinic and health center staff will be trained to ensure that staff offer holistic HIV and sexual and reproductive health services, including family planning, sexually transmitted infection diagnosis and treatment, and condom promotion, in a nonjudgmental and friendly manner. This training will include learning the SOPs, gaining hands-on experience by shadowing nurses at the same facility to understand clinic procedures, and receiving antistigma training. We will conduct a pilot study with 25 AGYW drawn from the study sampling cohort to finalize the SOPs.

### Cohort Recruitment and Sampling Frame

To select the study sites, we will identify six health centers or clinics that provide HIV services and cervical cancer screening in the Lusaka district. To establish a sampling frame from which to recruit participants for the randomized trial (described below), cohorts of HIV-/u and HIV+ AGYW will be recruited based on residence in the catchment areas of these clinics, which are the zones immediately surrounding the clinics. To avoid potential cross-contamination, we will select clinic zones that are not contiguous. All the clinics will be located within the greater Lusaka area, and each government clinic draws patients from specific catchment areas surrounding the clinic. We will therefore focus on ensuring that the clinic catchment areas do not overlap and are not adjacent to each other. We will conduct a discrete choice experiment concurrently with the recruitment of the AGYW for the sampling frame. The discrete choice experiment will be conducted to systematically evaluate preferences for HIV clinical care services (this information will also be used to further tailor the integrated wellness care clinic package of interventions, as appropriate). AGYW will be asked to choose among scenarios with varying combinations of key attributes relevant to HIV and other clinical care services (for example, service availability, wait time, operating hours, provider type, and protection of privacy). A key design consideration for the discrete choice experiment is to allow for both group-level and individual-level differences. We plan to perform subanalyses by age group and HIV status to assess differences by developmental stage and have ensured that the sample size of 1000 HIV-/u AGYW (approximately 330 in three age groups) and 800 HIV+ AGYW (approximately 400 in two age groups) will be adequate to perform the planned analysis [35-37]. The sample sizes required for the follow-on randomization were also taken into account for determining the size of the initial HIV-/u and HIV+ cohorts to establish the sampling frame.

To identify the HIV-/u cohort, we will map out each neighborhood and households within the clinic catchment area with the assistance of the local neighborhood health committees, the community liaisons attached to the clinics, and the outreach staff from the cervical cancer program. Peer navigators will visit each household, beginning with residential areas closest to the clinics, to identify eligible AGYW and recruit them to participate in the study. Participants will be notified of their rights, that questions regarding HIV status will be discussed, and that their refusal to participate will not affect their access to health care or other services. All AGYW aged ≥18 years will provide written informed consent in their preferred language, and written parental consent will be obtained for participants under 18 years of age, followed by written assent from these minors. We will include one AGYW per household and enroll AGYW on a continual basis until we reach our desired enrollment targets. Participants in the HIV-/u cohort must be female, be 10-20 years of age, and self-report their HIV status as negative or unknown (“unknown” defined as no HIV testing within the past 6 months).

We have selected the age group of 10-20 years because this is the highest-priority cohort and because prevention interventions are best delivered early to establish healthy behavior patterns before HIV exposure. Additional inclusion criteria include the participant not being pregnant, not suspecting she is pregnant, and not expressing a desire to become pregnant in the next 18 months; willing to sign a release for medical records (to obtain clinic data on service use); plans to reside in the same location for the next 18 months; and not been part of the other planned formative research activities. We will exclude pregnant AGYW because their motivations and health-seeking behaviors are likely to change during pregnancy, they require specialized antenatal care, and they are not eligible for the HPV vaccine that will be offered to the integrated wellness care clinic participants. All pregnant AGYW identified during the study will be offered assistance for obtaining care at appropriate antenatal clinics. We will recruit 160-170 adolescent girls from each clinic catchment area, with equal numbers in the 10-12, 13-15, and 16-20 year age ranges, for a total of 1000 girls.

For the HIV+ cohort, to maintain confidentially, we will request that health providers and community outreach staff at target clinics, along with staff from community-based testing centers, approach eligible HIV+ AGYW who reside in the clinic catchment area (identified through patient record review) to seek their consent to be contacted by the research team about the study. This preconsent process has been designed to be concise with as little detail of the study as possible to protect HIV+ participants’ confidentiality. Individuals administering the preconsent process will be able to give participants an overview of the study along with information on the methodology and research ethics. Should HIV+ AGYW accept, members of the research staff will contact them and administer a more detailed consent form to help inform the potential participants’ decision to participate. The use of community outreach staff will allow us to target AGYW who are HIV+ but not receiving treatment in addition to those who are actively engaged in care. Those who voluntarily indicate that they are HIV+ during recruitment of the HIV-/u cohort will also be given the opportunity to enroll in the HIV+ cohort if they are eligible. Participants who are female, 16-24 years of age, and diagnosed with HIV within the past 3 years (to target relatively recently diagnosed AGYW at the time of recruitment) will be eligible. Those who receive an HIV+ diagnosis during our 3-month recruitment time frame will also be included. In Zambia, sexual acts with adolescents younger than 16 years, even if consensual, are considered criminal. Given the possible negative repercussions that AGYW may experience should they be reported and our desire to maintain a trusting relationship with the communities that we wish to serve, we will not recruit adolescent girls aged ≤15 years for the HIV+ cohort. We will enroll 800 HIV+ AGYW, targeting a similar number (130-140) from each clinic zone with equal representation among those aged 16-20 years and 21-24 years. The additional inclusion criteria listed above will also be applied to the HIV+ cohort.

The systematic approach for establishing the sampling frame will provide discrete, nonduplicated individuals (the participant list will be updated daily); reduce selection bias by ensuring a more representative sampling for the cluster-randomized trial; and, if necessary, allow for adjustments in the random selection process, so that AGYW cohorts are similar across randomization arms.

### Randomized Controlled Trial

#### Cluster Randomization Study Design and Interventions

**Figure 2:**
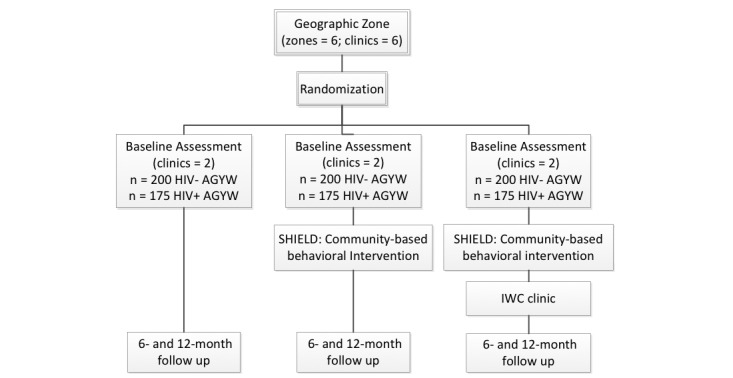
Experimental design and sample sizes. AGYW: adolescent girls and young women; SHIELD: Support for HIV Integrated Education, Linkages to Care, and Destigmatization; IWC: integrated wellness care.

**Figure 3:**
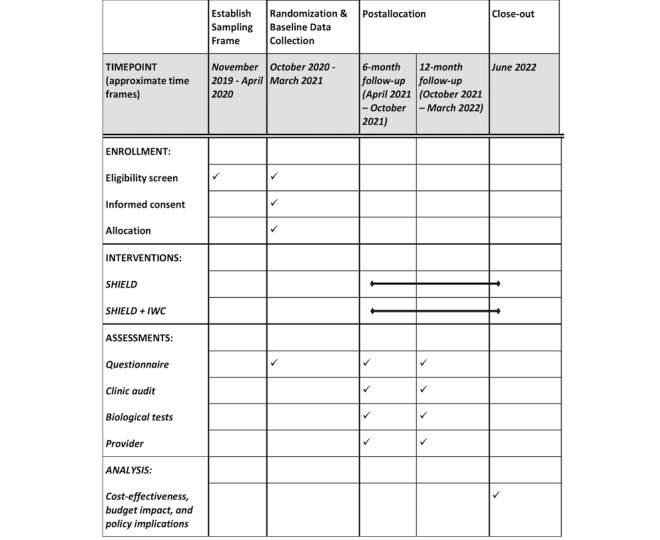
Standard Protocol Items: Recommendations for Interventional Trials (SPIRIT) flow diagram of the cluster-randomized controlled trial. IWC: integrated wellness care; SHIELD: Support for HIV Integrated Education, Linkages to Care, and Destigmatization.

The selected zones and their respective clinics will be randomized into three groups: zones with integrated wellness care clinics and SHIELD intervention (integrated wellness care+SHIELD), zones with only SHIELD intervention (SHIELD only), and control zones with no intervention at the clinic or community levels (usual care). This division will allow us to assess the impact of offering integrated wellness care clinics coupled with the SHIELD intervention compared with the standard of care and whether a similar impact can be achieved with control clinics when the SHIELD intervention is offered in the community. Figure 2 shows the trial design and group sample sizes. Figure 3 shows the study periods and timeline for the trial initiation, postallocation, and analysis. The SHIELD intervention, as described earlier, will begin with training sessions for AGYW and their families. This will be followed by peer navigator–facilitated youth clubs that will meet twice a month during the 12-month study time frame. Two peer navigators will be assigned to each clinic zone with the SHIELD intervention, and one peer navigator each will be assigned to the two control clinic zones to assist with data collection and follow-up activities. To avoid cross-contamination, each peer navigator will remain in their assigned zones for the study duration. The peer navigators in the zones with integrated wellness care clinics will also serve as community liaisons to support clinic-community linkages. The primary endpoint for the HIV-/u cohort is the proportion with any HIV testing at 12 months. For the HIV+ cohort, we plan to assess two primary endpoints: retention in care, measured as proportion with at least one visit during each 3-month period (over the 12-month follow-up), and viral load suppression measured as the proportion with undetectable viral load at 12 months. The secondary endpoints are the proportion with a decrease in HIV risk behavior, repeat HIV testing (each 6-month period), HIV infections diagnosed through HIV counseling and testing, linkages to HIV care, and adherence to ART. Table 1 presents the endpoint definitions and biological, self-report, and clinic audit measures collected at 6- and 12-month follow-ups. To assess the impact of the SHIELD intervention on the mediating outcomes, we will collect data from both AGYW and their caregivers (one family member identified by AGYW) from across all six clinic zones at baseline and at 6- and 12-month follow-ups. Additionally, we will track the health care service use by collecting clinic data at all six clinic sites. Information will be obtained from the clinical records at baseline and during the 6- and 12-month follow-ups by using prespecified abstraction forms. Specially trained staff will perform biological data collection, which will include HIV testing at 12 months for the HIV-/u group, viral load testing at 12 months for the HIV+ group, and pregnancy testing for both groups at baseline and 12 months.

**Table 1:** Measures and source at baseline, 6 months, and 12 months.

Constructs/measures		Instrument and specification	Source	Cohort
**Primary and secondary endpoints—HIV care continuum and HIV risk behavior**				
	HIV testing: proportion tested for HIV in the past 6 and 12 mo	Abstraction tool (serologic HCT^a^) and self-report confirmed via clinic audit; repeat HCT at 6-month intervals will be assessed	Clinic audit, self-report	HIV-/u^b^; 6 mo and 12 mo
	HIV early detection: proportion with HIV identified through voluntary testing	All participants will be tested to determine HIV status (rapid finger prick and follow-up per Zambian guidelines)	Biological	HIV-/u; 12 mo
	Linkage to care: proportion enrolled at an HIV clinic in ≤30 days; *ART initiated* in ≤*90 days*	Measured as days from testing HIV positive or from baseline when not on treatment (HIV+ but not on ART^c^)	Clinic audit	HIV-/u, HIV+^d^; 6 mo and 12 mo
	Retention in HIV care: proportion with at least one visit every 3 months	Prespecified abstraction tool to capture any HIV care–related visits over a 12-month period (clinic or dispensary visits)	Clinic audit	HIV+; 6 mo and 12 mo
	Adherence to ART: proportion filling prescriptions at least every 3 months	3-month supply of ART medications recommended by Zambian guidelines	Clinic audit	HIV+; 6 mo and 12 mo
	Viral load suppression: proportion with undetectable viral load	HIV plasma viral load tests using the Roche platform	Biological	HIV+; 6 mo and 12 mo
	HIV risk behavior: proportion with delay in first intercourse, reduction in sexual partners, increases in condom use	Demographic and health survey and AGEP^e^ instrument (measures will be based on previous studies by grant team, including AGEP)	Self-report	HIV-/u; baseline, 6 mo, and 12 mo
**Mediating outcomes, SHIELD^f^ intervention dose, and clinic-based services**				
	Self-efficacy	Measurement scale developed and tested for the AGEP	Self-report	HIV-/u and HIV+; baseline, 6 mo, and 12 mo (unless otherwise specified)
	Social support (AGYW^g^ report on family and peer interaction)	Medical Outcomes Study instrument previously used in Zambia [38]; AGEP PN^h^ and youth club feedback instrument	Self-report	HIV-/u and HIV+; baseline, 6 mo, and 12 mo (unless otherwise specified)
	Mental health	Strengths and Difficulties Questionnaire, youth version [39]	Self-report	HIV-/u and HIV+; baseline, 6 mo, and 12 mo (unless otherwise specified)
	HIV stigma, gender-based violence	AGEP instrument	Self-report	HIV-/u and HIV+; baseline, 6 mo, and 12 mo (unless otherwise specified)
	Unintended pregnancy	Tested at baseline and 12 mo	Urine test	HIV-/u and HIV+; baseline, 6 mo, and 12 mo (unless otherwise specified)
	Education modules (AGYW and family)	Number of modules completed	Study database	HIV-/u and HIV+; baseline, 6 mo, and 12 mo (unless otherwise specified)
	Youth club attendance	Number and proportion of meetings attended	Study database	HIV-/u and HIV+; baseline, 6 mo, and 12 mo (unless otherwise specified)
	Caregiver assessment	Parent Strengths and Difficulties Questionnaire and survey on AGYW support (to be developed)	Self-report	HIV-/u and HIV+; baseline, 6 mo, and 12 mo (unless otherwise specified)
	HPV^i^ vaccination	Proportion receiving 1, 2, or 3 doses (IWC^j^ clinic only)	Clinic audit	HIV-/u and HIV+; 6 mo and 12 mo
	Number of clinic visits	Proportion with visits at 6 mo and 12 mo; total number of visits	Clinic audit	HIV-/u and HIV+; 6 mo and 12 mo
	Sexual and reproductive health services provided	Family planning, sexually transmitted diseases, and condoms	Clinic audit	HIV-/u and HIV+; 6 mo and 12 mo
**PNs and clinic staff feedback on AGYW HIV care**				
	PNs	PN feedback instrument (to be developed)	Self-report	Baseline, 6 mo, and 12 mo
	Clinic staff attitudes and feedback	10 staff interviewed at each clinic; modified Nyblade instrument	Self-report	Baseline, 6 mo, and 12 mo
**Baseline data for AGYW**				
	Demographics and socioeconomic status	AGEP baseline instrument	Self-report	All AGYW

^a^HCT: HIV counseling and testing.

^b^HIV-/u: HIV-negative/unknown.

^c^ART: antiretroviral therapy.

^d^HIV+: HIV-positive.

^e^AGEP: Adolescent Girl Empowerment Program.

^f^SHIELD: Support for HIV Integrated Education, Linkages to Care, and Destigmatization.

^g^AGYW: adolescent girls and young women.

^h^PN: peer navigator.

^i^HPV: human papillomavirus.

^j^IWC: integrated wellness care.

#### Selection of Adolescent Girls and Young Women and Sample Size Determination

In each clinic zone, we will use stratified random sampling, using age-group strata drawn from the sampling frame previously established, to select AGYW for the HIV-/u and HIV+ study cohorts (integrated wellness care pilot study participants will be excluded). The selection criteria will remain the same as the sampling frame recruitment. Those who become pregnant (confirmed by urine test at intake) will be excluded, and those who become HIV seropositive (self-reported) will be assigned to the HIV+ cohort if they meet the age criteria; we will oversample by 20% in each age subgroup to account for ineligibility. Because we will have an established sampling frame, we will review the AGYW characteristics in each clinic zone to determine whether a more complex selection process is warranted to control for potential differences in characteristics between AGYW. The sample size was calculated assuming conventional specifications (power=.80, alpha=.05, two-sided tests) with an intracluster correlation coefficient (ICC) close to 1 and based on the primary endpoints. The justification for using an ICC of ~1 is supported by our knowledge of the six selected local government clinics and their surrounding communities (clusters). The clinics all provide the same standardized clinical services in similar facilities and serve a generally homogeneous catchment population of low-income individuals. Additionally, our analysis will be conducted at the individual level, which will allow us to control for any unanticipated variation at the individual or cluster level.

The sample sizes allow for the detection of a 15-percentage point improvement in the primary endpoints, which is our minimum threshold to consider the intervention to be successful. For the HIV-/u cohort, the current rate of HIV testing is estimated to be about 40%. With a sample size of 200 per randomization groups, we will be able to detect a 13%-14% difference. The grant team achieved a 90% retention rate at 12 months in a recent study of AGYW [18]; with a loss of 10% of the cohort during our follow-up in this study, we will still have the statistical power for a minimum detectable difference of 15% in HIV testing. For the HIV+ cohort, both retention in care and viral load suppression is estimated to be 70%-75% among AGYW. With a sample size of 175 per randomization group, we will be able to detect a minimum difference of 15% even with a 10% loss to follow-up. We will enroll 600 HIV-/u AGYW (10-20 years of age) and 525 HIV+ AGYW (16-24 years of age) across the three randomization groups.

#### Hypotheses on Intervention Effect

We will test the following hypotheses:

- HIV-/u AGYW from zones randomized to integrated wellness care+SHIELD will have higher HIV testing than AGYW in zones randomized to SHIELD only or usual care.
- HIV+ AGYW from zones randomized to integrated wellness care+SHIELD will have higher retention in care and viral load suppression than AGYW in zones randomized to SHIELD only or usual care.
- HIV+ AGYW from zones randomized to integrated wellness care+SHIELD will receive more timely linkages to care than AGYW in zones randomized to SHIELD only or usual care.
- AGYW from zones randomized to integrated wellness care + SHIELD or SHIELD only will have improved self-efficacy and social support and reduced HIV risk behaviors compared with AGYW from zones randomized to usual care.

#### Analysis of Hypotheses

The testing of the primary hypotheses will be done by intent to treat, and we will examine the effects across the three randomization groups. Given the need to consider the influence of the cluster randomization by zone, we will use generalized estimating equation models to estimate the effects of the randomization groups and will assess the need to apply small sample correction using approaches suggested by the National Institutes of Health Care Systems Collaboratory Biostatistics and Design Core [40]. If the Hausman assumption of correlation between the random and fixed effects is violated, then we will include fixed effects representing cluster identification. We will adjust for baseline covariates, including sociodemographic factors and behavioral risks, should the initial descriptive analyses suggest differences in the distribution of these factors across study randomization groups. We will also explore the use of propensity scores to control for systematic differences between the groups. In further analyses, we will examine the potential mediating and moderating roles of key behavioral, social, and structural factors hypothesized to influence HIV testing and adherence along the HIV care continuum (eg, social support and self-efficacy), as shown in Figure 1. The specific measures, along with the instruments and specifications, that will be used are shown in Table 1. We will test the “dose” of the SHIELD training received as a covariate in these models.

Secondary analysis will follow the same approach. As appropriate, we will perform analyses separately or pooled together for the HIV+ and HIV-/u cohorts. A key aspect of interest in this study is the developmental stage of the AGYW; using age as a proxy in our multivariate analysis, we will determine the differential effect of the integrated wellness care clinic and SHIELD intervention on outcomes reported by age group and HIV status. In subsequent analyses, we will also consider both medium-term outcomes (within the first 6 months after study enrollment) and longer-term outcomes (within the first 12 months after study enrollment). Additionally, for measures where we have baseline, 6-month, and 12-month follow-up data (for example, self-efficacy and other mediating outcomes for AGYW and caregiver), we will perform difference-in-difference analysis, which will allow for comparisons between and across the study arms. We will also report all mediating factors, caregiver outcomes, and integrated wellness care clinic utilization metrics stratified by age and HIV status to facilitate subgroup analysis. Importantly, the changes along the HIV care cascade, including HIV testing, linkage to care, retention in care, ART adherence, and viral load suppression, will be documented to identify potential differential impacts of the interventions. This analysis is critical to assist in further tailoring the SHIELD training and integrated wellness care clinic services.

#### Cost-Effectiveness, Budget Impact, and Policy Implications

We will use a previously validated instrument, The Cost Assessment Tool [41], to collect resource use information on the interventions. Our main goal is to estimate the implementation cost of the integrated wellness care and the SHIELD interventions from the program perspective, but we will also derive the start-up costs related to developing the interventions to inform future adaption of these interventions to other settings. We will estimate labor hours by prospectively tracking time spent by each project staff member on a predefined set of activities (individuals will report their time monthly) and use hourly wage to calculate costs. We will also document the expenditure on nonlabor resources. Using standard economics methodology [42,43], we will explore economies of scale that can be achieved during scale-up. The cost information, along with the impact of effectiveness of the integrated wellness care and SHIELD interventions, will be used as inputs in previously validated models [44]. We will evaluate the tradeoff, or return on investment, between investing in HIV prevention and lowering HIV treatment cost over the long term. The usual care (base case) will be compared to the long-term effectiveness of including the SHIELD community-based interventions with and without the integrated wellness care clinic-based intervention to address barriers along the continuum of care. We will report the projected decrease in HIV incidence, the projected increase in community-level viral load suppression, and the incremental cost per quality-adjusted life years. We will conduct policy simulations to assess the impact of scaling up the interventions to the population level; perform sensitivity analysis, varying the range of effectiveness and cost estimates; and generate potential best- and worst-case scenarios. We will create tornado and spider diagrams to display this uncertainty assessments graphically to policy makers. This cost-effectiveness analysis will be complemented by a budget analysis, which will identify the annual financial outlays that will be required to implement the interventions during various phases in the scale-up process.

## Results

Data collection from formative research will be completed in March 2020. The cluster-randomized trial is expected to begin in 2020. Analysis will be performed to test the hypotheses indicated, and additional analysis will be conducted on the cost-effectiveness, budget impacts, and policy implications of the interventions. Results of this study will be shared with the research community and the public at large through conference presentations and publication in peer-reviewed journals. Results are expected in mid-2023.

## Discussion

### Limitations and Approaches to Minimize Bias

First, although cluster randomization reduces contamination across study arms, it increases the risk that clinics and individuals in each arm may differ at baseline. The unique feature of this study is that we will create a sampling frame and will therefore be able to assess individual-level baseline differences before randomization. The sampling frame will also allow us to adjust our sampling process or *a priori* include propensity score weighting methods during analysis to match individuals with similar characteristics. Additionally, through our planned situational analysis, we will explore clinic characteristics to select clinics that are similar.

Second, data could be missing because of nonresponse as well as study attrition. All AGYW will be assigned to a peer navigator who will ensure regular contact with the AGYW during the study period. We will also employ rigorous field data collection practices, including training data collectors, developing protocols, and monitoring fidelity on a continual basis. Using this approach, on the basis of a recent study among Zambian AGYW by the study team, we expect that retention will be 90% over the 12-month follow-up period [18]. Furthermore, we will address any missing data by including demographic covariates that will serve as proxies for dropout and by conducting sensitivity analyses.

### Policy Implications

The knowledge gained in this study will address a critical gap in our understanding of effective public health interventions to improve engagement of AGYW along the HIV care continuum. It will test community- and clinic-based interventions to address key barriers to engagement in care at the individual, interpersonal, and clinic levels including HIV knowledge, stigma, social support, and the need for youth-friendly services. If successful, the proposed interventions will improve HIV testing, retention in care, and treatment adherence among AGYW and will contribute to meeting the 90-90-90 and 95-95-95 targets, reducing secondary transmission and improving the quality of life of AGYW affected by HIV. Successful implementation of this multilevel intervention to establish a comprehensive care continuum for HIV-affected AGYW will provide a strong evidence base for expanding integrated HIV and sexual and reproductive health services for AGYW both nationally and within sub-Saharan Africa.

## Appendix

Multimedia Appendix 1 Peer-reviewer report from the NIH.

## Abbreviations

AGEP: Adolescent Girl Empowerment Program
AGYW: adolescent girls and young women
ART: antiretroviral therapy
HCT: HIV counseling and testing
HIV-/u: HIV-negative or unknown
HIV+: HIV-positive
HPV: human papillomavirus
ICC: intracluster correlation coefficient
IWC: integrated wellness care
PN: peer navigator
SHIELD: Support for HIV Integrated Education, Linkages to Care, and Destigmatization
SOP: standard operating procedure

## References

[1] (2016). UNAIDS.

[2] Wong VJ, Murray KR, Phelps BR, Vermund SH, McCarraher DR (2017). Adolescents, young people, and the 90-90-90 goals: a call to improve HIV testing and linkage to treatment. AIDS.

[3] Kooma E, Chinyonga J, Banda R, Nondo R, Sakalunda L, Muchaya T, Chiiya H (2016). Assessment of Core Capacities for the Implementation of International Health Regulations (2005) at selected Points of Entry (POEs). A case for Southern, Western and Lusaka Provinces of Zambia. IJAR.

[4] (2017). UNAIDS.

[5] (2016). World Health Organization.

[6] Hudelson C, Cluver L (2015). Factors associated with adherence to antiretroviral therapy among adolescents living with HIV/AIDS in low- and middle-income countries: a systematic review. AIDS Care.

[7] Sam-Agudu NA, Folayan MO, Ezeanolue EE (2016). Seeking wider access to HIV testing for adolescents in sub-Saharan Africa. Pediatr Res.

[8] (2016). PEPFAR: Latest Global Results.

[9] Kalibala S, Mulenga D (2011). Situation assessment of the HIV response among young people in Zambia.

[10] Abdool Karim Q, Baxter C, Birx D (2017). Prevention of HIV in Adolescent Girls and Young Women: Key to an AIDS-Free Generation. J Acquir Immune Defic Syndr.

[11] (2015). Lusakatimes.com.

[12] Zambia DREAMS overview.

[13] Govindasamy D, Ferrand RA, Wilmore SM, Ford N, Ahmed S, Afnan-Holmes H, Kranzer K (2015). Uptake and yield of HIV testing and counselling among children and adolescents in sub-Saharan Africa: a systematic review. J Int AIDS Soc.

[14] Reddy EA, Agala CB, Maro VP, Ostermann J, Pence BW, Itemba DK, Safley D, Yao J, Thielman NM, Whetten K (2016). Test site predicts HIV care linkage and antiretroviral therapy initiation: a prospective 3.5 year cohort study of HIV-positive testers in northern Tanzania. BMC Infect Dis.

[15] Bauman LJ, Braunstein S, Calderon Y, Chhabra R, Cutler B, Leider J, Rivera A, Sclafane J, Tsoi B, Watnick D (2013). Barriers and facilitators of linkage to HIV primary care in New York City. J Acquir Immune Defic Syndr.

[16] MacPherson P, Lalloo DG, Webb EL, Maheswaran H, Choko AT, Makombe SD, Butterworth AE, van Oosterhout JJ, Desmond N, Thindwa D, Squire SB, Hayes RJ, Corbett EL (2014). Effect of optional home initiation of HIV care following HIV self-testing on antiretroviral therapy initiation among adults in Malawi: a randomized clinical trial. JAMA.

[17] (2016). Global Consultation on Lessons from Sexual and Reproductive Health Programming to Catalyse HIV Prevention for Adolescent Girls and Young Women. 2nd edition.

[18] Austrian K, Hewett P, Soler-Hampejsek E, Bozzani F, Behrman J, Digitale J (2016). Adolescent Girls Empowerment Programme: Research and evaluation mid-term technical report.

[19] Clay S, Chonta M, Chiiya C, Mackworth-Young C, Bond V, Stangl A (2018). Tikambisane ‘Let’s Talk to Each Other’: A 6-session support group curriculum for adolescent girls living with HIV in Zambia.

[20] Welbourn A (1995). Stepping stones. A training package on HIV/AIDS, communication and relationship skills (Strategies for Hope Training, Series 1).

[21] Skevington SM, Sovetkina EC, Gillison FB (2013). A systematic review to quantitatively evaluate 'Stepping Stones': a participatory community-based HIV/AIDS prevention intervention. AIDS Behav.

[22] Steward WT, Sumitani J, Moran ME, Ratlhagana M, Morris JL, Isidoro L, Gilvydis JM, Tumbo J, Grignon J, Barnhart S, Lippman SA (2018). Engaging HIV-positive clients in care: acceptability and mechanisms of action of a peer navigation program in South Africa. AIDS Care.

[23] Winskell K, Miller KS, Allen KA, Obong'o CO (2016). Guiding and supporting adolescents living with HIV in sub-Saharan Africa: The development of a curriculum for family and community members. Children and Youth Services Review.

[24] MacKenzie RK, van Lettow M, Gondwe C, Nyirongo J, Singano V, Banda V, Thaulo E, Beyene T, Agarwal M, McKenney A, Hrapcak S, Garone D, Sodhi SK, Chan AK (2017). Greater retention in care among adolescents on antiretroviral treatment accessing. J Int AIDS Soc.

[25] (2015). Promising practices: one-stop adolescent shop: delivering adolescent-friendly sexual and reproductive health, HIV and TB services.

[26] Mburu G, Ram M, Oxenham D, Haamujompa C, Iorpenda K, Ferguson L (2014). Responding to adolescents living with HIV in Zambia: A social–ecological approach. Children and Youth Services Review.

[27] Fwemba I, Musonda P (2017). Modelling adverse treatment outcomes of HIV-infected adolescents attending public-sector HIV clinics in Lusaka. Public Health.

[28] Parham GP, Mwanahamuntu MH, Sahasrabuddhe VV, Westfall AO, King KE, Chibwesha C, Pfaendler KS, Mkumba G, Mudenda V, Kapambwe S, Vermund SH, Hicks ML, Stringer JS, Chi BH (2010). Implementation of cervical cancer prevention services for HIV-infected women in Zambia: measuring program effectiveness. HIV Ther.

[29] Mwanahamuntu MH, Sahasrabuddhe VV, Pfaendler KS, Mudenda V, Hicks ML, Vermund SH, Stringer JSA, Parham GP (2009). Implementation of 'see-and-treat' cervical cancer prevention services linked to HIV care in Zambia. AIDS.

[30] Liu FW, Vwalika B, Hacker MR, Allen S, Awtrey CS (2012). Cervical cancer and HPV vaccination: Knowledge and attitudes of adult women in Lusaka, Zambia. J Vaccines Vaccin.

[31] Subramanian S, Nyambe N, Hoover S, Pender L, Chibwesha C, Parham G (2017). Insights into the delivery of HPV vaccination, HIV services and cervical cancer screening in Zambia (RTI Working Paper).

[32] Bandura A (1994). Social cognitive theory exercise of control over HIV infection. Preventing AIDS: Theories and Methods of Behavioral Interventions, DiClemente RJ, Peterson J. editors.

[33] Kamala BA, Rosecrans KD, Shoo TA, Al-Alawy HZ, Berrier F, Bwogi DF, Miller KS (2017). Evaluation of the Families Matter! Program in Tanzania: An Intervention to Promote Effective Parent-Child Communication About Sex, Sexuality, and Sexual Risk Reduction. AIDS Educ Prev.

[34] Miller KS, Lasswell SM, Riley DB, Poulsen MN (2013). Families matter! Presexual risk prevention intervention. Am J Public Health.

[35] Reed Johnson F, Lancsar E, Marshall D, Kilambi V, Mühlbacher A, Regier DA, Bresnahan BW, Kanninen B, Bridges JFP (2013). Constructing experimental designs for discrete-choice experiments: report of the ISPOR Conjoint Analysis Experimental Design Good Research Practices Task Force. Value Health.

[36] Hall J, Kenny P, King M, Louviere J, Viney R, Yeoh A (2002). Using stated preference discrete choice modelling to evaluate the introduction of varicella vaccination. Health Econ.

[37] Lancsar E, Louviere J (2008). Conducting Discrete Choice Experiments to Inform Healthcare Decision Making. PharmacoEconomics.

[38] Menon A, Glazebrook G (2013). Randomized control trial to evaluate yoga-based peer support group for human immunodeficiency virus (HIV) positive Zambian adolescents. Journal of AIDS and HIV Research.

[39] Menon A, Glazebrook C, Campain N, Ngoma M (2007). Mental health and disclosure of HIV status in Zambian adolescents with HIV infection. JAIDS.

[40] Cook AJ, Delong E, Murray DM, Vollmer WM, Heagerty PJ (2016). Statistical lessons learned for designing cluster randomized pragmatic clinical trials from the NIH Health Care Systems Collaboratory Biostatistics and Design Core. Clin Trials.

[41] Subramanian S, Ekwueme DU, Gardner JG, Trogdon J (2009). Developing and testing a cost-assessment tool for cancer screening programs. Am J Prev Med.

[42] Trogdon JG, Ekwueme DU, Subramanian S, Crouse W (2014). Economies of scale in federally-funded state-organized public health programs: results from the National Breast and Cervical Cancer Early Detection Programs. Health Care Manag Sci.

[43] Subramanian S, Tangka FKL, Hoover S, Degroff A, Royalty J, Seeff LC (2011). Clinical and programmatic costs of implementing colorectal cancer screening: evaluation of five programs. Eval Program Plann.

[44] Subramanian S (2017). Cost-effectiveness of integrated HPV vaccination and screening: Impact on HIV and non-HIV infected women. http://aorticconference.org/wp-content/uploads/2017/10/2017-AORTIC-Abstracts.pdf.

