# Supplementary material for: Integrated Care Delivery for HIV Prevention and Treatment in Adolescent Girls and Young Women in Zambia: Protocol for a Cluster-Randomized Controlled Trial
Source: JMIR Res Protoc. 2019 Oct 3;8(10):e15314. doi: 10.2196/15314 (PMC6797972; doi:10.2196/15314)
Supplement: Multimedia Appendix 1 [file resprot_v8i9e15314_app1.pdf]

SUMMARY STATEMENT
(Privileged Communication)

PROGRAM CONTACT:
Bill Kapogiannis
(301)402-0698
kapogiannisb@mail.nih.gov

Release Date: 05/04/2018
Revised Date:

Application Number: 1 UG3 HD096908-01

Principal Investigator

SUBRAMANIAN, SUJHA

Applicant Organization: RESEARCH TRIANGLE INSTITUTE

Review Group: ZHD1 DSR-N (52)
National Institute of Child Health and Human Development Special Emphasis Panel AIDS - EXP. REV.

Meeting Date: 04/09/2018
Council: MAY 2018
Requested Start: 07/01/2018
RFA/PA: HD18-032
PCC: MPIDB-WK
Dual IC(s): MD

Project Title: Developing and Testing a Multi-level Package of Interventions for an Integrated Care Delivery Model of HIV Prevention and Treatment Targeting Adolescent Girls in Zambia
SRG Action: Impact Score:32
Next Steps: Visit https://grants.nih.gov/grants/next_steps.htm
Human Subjects: 30-Human subjects involved - Certified, no SRG concerns
Animal Subjects: 10-No live vertebrate animals involved for competing appl.
Gender: 1A-Both genders, scientifically acceptable
Minority: 1A-Minorities and non-minorities, scientifically acceptable
Children: 1A-Both Children and Adults, scientifically acceptable

Project Year	Direct Costs Requested	Estimated Total Cost
1	559,117	841,728
2	852,666	1,283,654
3	1,020,750	1,536,698
4	824,964	1,241,950
5	406,030	611,262
TOTAL	3,663,527	5,515,291

ADMINISTRATIVE BUDGET NOTE: The budget shown is the requested budget and has not been adjusted to reflect any recommendations made by reviewers. If an award is planned, the costs will be calculated by Institute grants management staff based on the recommendations outlined below in the COMMITTEE BUDGET RECOMMENDATIONS section.

1UG3HD096908-01 SUBRAMANIAN, SUJHA

RESUME AND SUMMARY OF DISCUSSION: This research grant application proposes to test a multilevel package of interventions to connect adolescent girls and young women (AGYW) with health care resources to provide regular HIV testing and adherence to antiviral treatment. The impact of this work would not only identify at risk and positive individuals, but it would ensure adequate health care services and support for adolescent girls and young women in Zambia. Conducting work in the age group of 15-24 years old is significant, because it has the potential to increase testing and medication adherence. This directly addresses the PATCH3 criteria, and it builds on an existing infrastructure. This highly skilled investigative team of researchers from strong programs has complementary expertise and experience to successfully complete the proposed work. They have experience in behavioral science, women's health, infectious disease, and adolescent care. The proposed work is innovative by using established collaborations and infrastructure; by using Discrete Choice Experiments; and by using the SHIELD community-based tool. The approach is well-developed and reasonable; appropriately uses a cluster randomized trial; has a feasible time line for the phases of the study as well as a reasonable participant recruitment plan. It also addresses cost effectiveness analysis using validated models and utilizes rigorous stage testing of tools and concepts. The research environment is exceptional with sufficient resources and established programs to conduct this work. Weaknesses of the proposal include the following: questionable sustainability after the award ends; lack of justification for combining and testing the SHIELD and IWC interventions; questionable analytical design; concerns regarding scalability of the SHIELD intervention; an unconvincing argument how IWC clinics differ from ART clinics; and a missed opportunity by not addressing men and HIV. Additionally, there is no information regarding how the team will interact or meeting frequency. Finally, there is no preliminary data regarding enrollment or previous successful enrollment information. Overall reviewer enthusiasm was medium to high impact with the application scoring in the range of good to excellent.

DESCRIPTION (provided by applicant): Zambia has one of the highest incidences of HIV in the world, and adolescent girls and young women (AGYW) are particularly affected. Prior studies and initiatives attempted to provide youth-friendly services through adolescent antiretroviral therapy (ART) clinics that offer a range of services, but these clinics have faced challenges because loss of privacy and the stigma associated with HIV. To address this gap, the Zambian Ministry of Health, RTI International, the Population Council, and the University of North Carolina are collaborating to create an integrated wellness care (IWC) delivery model that targets all HIV-affected AGYW, both those that are HIV negative or do not know their status (HIV-/u) and those that are HIV positive (HIV+). We will test a multilevel package of interventions to connect AGYW with a source of regular care to provide a sustainable platform for successful implementation of regular HIV testing and support for linkage to care, retention in care, and adherence to antiviral treatment. We propose to pursue the following aims: UG3–1. Engage stakeholders by establishing community and youth advisory boards, conduct formative research, adapt modules for behavioral intervention, and develop data collection instruments. UG3–2. Recruit AGYW to establish a sampling frame and perform a discrete choice experiment to systematically evaluate preferences for HIV clinic-based services (1,000 HIV-/u AGYW aged 10 to 20 years and 800 HIV+ AGYW aged 16 to 24 years) to ensure the IWC clinic is tailored to AGYW's needs. UG3–3. Develop standard operating procedures (SOPs) for IWC clinic service delivery and structure, identify and train IWC clinic staff, engage the Youth Advisory Board to create a youth-friendly environment, and conduct a pilot study to evaluate implementation processes (25 AGYW). UH3–1. Assess efficacy at 6 and 12 months of the multilevel interventions at the individual (HIV knowledge, self-efficacy), interpersonal (social support, stigma reduction), and health system (IWC clinic) levels on HIV testing, retention in care, and viral load suppression using a cluster randomized design. UH3–2. Conduct in-depth interviews with clinical staff and peer navigators at 12 months to obtain feedback on the integrated care delivery model to assess sustainability, document best practices, and update SOPs to support scaling up of integrated services for AGYW. UH3–3. Perform cost-effectiveness and budget

analysis to evaluate and describe impact along the HIV care continuum and disseminate findings to national partners and the international community. This integrated service delivery model, if successful, can also serve as a platform to implement additional preventive services, including pre-exposure prophylaxis (PrEP) for high-risk AGYW, which is currently under consideration by the government.

PUBLIC HEALTH RELEVANCE: Zambia has one of the highest incidences of HIV in the world, and adolescent girls and young women (AGYW) are a particularly affected group because of their social and economic vulnerability. The overall goal of this study is to test a multilevel package of interventions at the community and health system levels to connect AGYW with a source of regular care to provide a sustainable platform for successful implementation of regular HIV testing and adherence to antiviral treatment. This approach will avoid siloes and provide a comprehensive HIV care continuum with a holistic and integrated health care delivery approach that is recommended by the Zambian HIV guidelines.

CRITIQUE 1

Significance: 3
Investigator(s): 1
Innovation: 2
Approach: 3
Environment: 1

Overall Impact: This high impact application addresses low testing and poor adherence to ART therapy among AGYW in Zambia by seeking to adapt a successful cervical cancer screening program to integrate services such as providing the HPV vaccine and HIV testing to young women, and linking seroconverters to care. If successful, this could serve as an important approach to identify HIV-infected and at-risk AGYW for additional services. Using the IWC clinics as an entry point to testing and care, and the DCE are innovative approaches. The team is led by a highly accomplished and well-trained investigator, Dr. Subramanian, and she will be assisted by an excellent team of collaborators with well-defined roles. A key weakness that raised concerns is the SHEILD intervention and the potential for its sustainability beyond the study period. Another concern is with the discrete choice experiments which will use the same sample that participates in the randomized trial. Will this introduce bias in the findings?

An application does not need to be strong in all categories to be judged likely to have major scientific impact. For example, a project that by its nature is not innovative may be essential to advance a field.

1. Significance:

Strengths

- The scientific premise for this study is strong because while HIV prevalence is high among adolescent girls and young women (AGYW) aged 15-24 in Zambia, they are also less likely to test and adhere to ART therapy than older women.
- This application seeks to adapt a successful cervical cancer screening program to integrate services such as providing the HPV vaccine and HIV testing to young women. If successful, this could serve as an important approach to identify HIV-infected and at-risk AGYW for additional services.

Weaknesses

- The potential for the sustainability of the SHIELD intervention is unclear. Previous studies have found that programs that include youth clubs are not sustainable beyond the study period or when donor funding ends. How will this be addressed in the study?

2. Investigator(s):

Strengths

- Dr. Subramanian is an accomplished investigator who is very well suited to lead the study.
- An excellent team of seasoned collaborators with commendable achievements, expertise, and skills.

Weaknesses

- None noted.

3. Innovation:

Strengths

- The approach to use integrated wellness clinics (IWCs)to provide HIV testing, HIV treatment (in coordination with the HIV clinic and dispensary in the same facility), HPV vaccination, and other SRH services to AGYW is innovative.
- Discrete Choice Experiments is an innovative method to understand how AGYW value services and attributes.

Weaknesses

- Justification is not provided for testing the combination of the SHIELD and IWC interventions.

4. Approach:

Strengths

- The timeline for the UG3 and UH3 and the Transition Milestones phases are well-reasoned, feasible, and appropriate.
- The strategy for UG3-1 is well developed and reasonable.
- Focus on AGYW for the proposed study is appropriate and justified.
- Establishing a sampling frame will lead to enrollment of adequate numbers of AGYW.
- Assigning peer navigators to specific zones will help insure recruitment of discrete, non-duplicated subjects.

Weaknesses

- The same sample that participates in the DCE will also participate in the randomized trial. Will this introduce bias in the findings?
- It is not clear from the application the goal of the SHIELD intervention separate from that of the IWC.
- Rigor of the analytical design is in question because the application does not adequately describe how the separate effects of the SHIELD intervention on AGYW will be assessed i.e. direct effect of the intervention on AGYW and indirect effect on AGYW through the intervention effects on caregivers and other family members.

- Scalability and sustainability, particularly of the SHIELD intervention is doubtful. The likelihood of scalability and sustainability is greater for the IWC approach.

5. Environment:

Strengths

- RTI, Population Council, and UNC-Zambia offer exceptional environments that will ensure success.

Weaknesses

- None noted.

— Responses for Protections for Minor Participants, Human Subjects, Vertebrate Animals, and

— Biohazards:

— A response for Inclusion of Women, Minorities and Children **is required from reviewers** for applications proposing Human Subjects Research

Protections for Minor Participants:

- Adequate plans are in place to enroll minors and for referral of seroconverters and high-risk participants.
- Adequate plans to protect participant information.

Protections for Human Subjects:

Data and Safety Monitoring Plan (Applicable for Clinical Trials Only):

Inclusion of Women, Minorities and Children:

- Sex/Gender:
- Race/Ethnicity:
- For NIH-Defined Phase III trials, Plans for valid design and analysis:
- Inclusion/Exclusion of Children under 18:

Vertebrate Animals:

Biohazards:

Applications from Foreign Organizations:

Select Agent Research:

Resource Sharing Plans:

Authentication of Key Biological and/or Chemical Resources:

Budget and Period of Support:

Recommended budget modifications or possible overlap identified:

Additional Comments to Applicant (Optional):

CRITIQUE 2

Significance: 2
Investigator(s): 2
Innovation: 3
Approach: 3
Environment: 1

Overall Impact: 3

An application does not need to be strong in all categories to be judged likely to have major scientific impact. For example, a project that by its nature is not innovative may be essential to advance a field.

1. Significance:

Strengths

- Directly addresses the PATCH3 targets, in particular, challenges with engaging and providing supportive care to young women.
- The AIMS are ambitious and potentially transformative.
- The SHIELD, community-based intervention appears particularly appealing.

Weaknesses

- The investigators first acknowledge the problems with "HIV clinics' and stigma, but then propose nesting the work in existing HIV clinics. In their defense, they will compare with community-based interventions.

2. Investigator(s):

Strengths

- Very strong investigators, led by Dr. Subramanaian. They emanate from strong programs at RTI, Population Council, UNC, and Zambian MOH. – A wisely chosen set of collaborators.
- Key experience and accomplishments in critical, central areas of the project.

Weaknesses

3. Innovation:

Strengths

- The SHIELD community-based tool appears innovative and well thought out.

Weaknesses

- This is both a strength and weakness: all elements of the project have essentially been created and are in the field to some extent. While there will be some tool building, this is essentially implementation of existing tools.

4. Approach:

Strengths

- Partnership between strong NGOs and the Zambian MOH - bodes well for future implementation if successful.
- Experts in clinical implementation, social-behavioral concepts, and stigma.
- Rigorous UH stage testing of tools and concepts.
- Interesting linkage of core implementation science (PEPFAR/USAID) and behavioral implementation research (NIH/CDC).
- Nice study of cost and cost effectiveness at the UH3 level – worry that this won't be scalable.

Weaknesses

- A convincing argument has not been provided that the IWC clinics are sufficiently different from ART clinics.
- Will the program primarily recruits those open to care and interventions and not be fully representative of the community at large? No analysis of those who refuse participation which may be very important for future planning and programmatic design. Consider dressing this more formally in the UG3-2 sampling frame establishment.
- The proposed DCE selections seem obvious – won't there be a groundswell towards no wait time, high level privacy, flexible hours, and very low to no cost of care?
- Differing PN support for the zone clusters may affect outcomes.
- Encourage 6 month as well as 12-month VL outcomes (Exhibit 8).
- Addresses women only – very important, but misses half of the critical population.

5. Environment:

Strengths

- Very strong environment with established programmatic infrastructure.
- Linkage to local governing and oversight bodies.

Weaknesses

— Responses for Protections for Minor Participants, Human Subjects, Vertebrate Animals, and

— Biohazards:

— A response for Inclusion of Women, Minorities and Children **is required from reviewers** for applications proposing Human Subjects Research

Protections for Minor Participants:

- Appropriate

Protections for Human Subjects:

- Appropriate

Data and Safety Monitoring Plan (Applicable for Clinical Trials Only):

- Appropriate

Inclusion of Women, Minorities and Children:

- Sex/Gender: Appropriate for study goals
- Race/Ethnicity: Appropriate
- For NIH-Defined Phase III trials, Plans for valid design and analysis:
- Inclusion/Exclusion of Children under 18: Appropriate

Vertebrate Animals:

- Not Applicable

Biohazards:

- Not applicable

Applications from Foreign Organizations:

- Appropriate

Select Agent Research:

- Not applicable

Resource Sharing Plans:

- Appropriate

Authentication of Key Biological and/or Chemical Resources:

- Not applicable

Budget and Period of Support:

Recommended budget modifications or possible overlap identified:

- Appropriate

Additional Comments to Applicant (Optional):

CRITIQUE 3

Significance: 3
Investigator(s): 2
Innovation: 3
Approach: 4
Environment: 2

Overall Impact:

This is an interesting study targeting adolescent girls and young women in Zambia to increase HIV testing, linkage to care, retention in care, and viral suppression by developing Integrated Wellness Clinics based on previous cervical cancer screening programs. This is a collaborative effort by the Zambian Ministry of Health, RTI International, The Population Council, and the University of North Carolina. The main goals are to increase HIV testing by HIV negative and unknown youth and for those living with HIV to keep them engaged in care and support adherence/viral suppression. This study will take advantage of a HPV vaccine role out. This is a strong proposal from a talented and experienced group of investigators that will expand on existing infrastructure and collaborations within the Zambian Ministry of Health. It would be strengthened if the proposal contained preliminary date or information on the initial cervical cancer screening study to evaluate potential enrollment and feasibility of enrolling 1800 individuals in 1 year.

An application does not need to be strong in all categories to be judged likely to have major scientific impact. For example, a project that by its nature is not innovative may be essential to advance a field.

1. Significance:

Strengths

- Targeting high risk population of adolescent girls and young women in Zambia.
- Targeting populations living with and without HIV
- Expanding on existing infrastructure and working with the national department of health.
- Analysis of efficacy as well as sustainability and cost effectiveness

Weaknesses

- The intervention is only targeting females; therefore, does not address the male population or structural processes involved in new infections although this still fits within the RFA.
- No mention of prevention services for those who are HIV negative other than uptake of HIV testing. This seems like a missed opportunity to offer expanded prevention services or PrEP, or guage PrEP interest if not yet available in Zambia.

2. Investigator(s):

Strengths

- Collaborations from UNC, the Zambian Ministry of health, and RTI to inform national guidelines and affect policy change and implementation
- Established research collaboration and working with this population
- Dr. Subramanian has experience recruiting young women in Cervical Cancer screening R01
- Team includes behavioral scientists, women's health, infectious disease, adolescent care

Weaknesses

- The research team is large and quite diverse. The proposal does not indicate how the investigators will interact and how frequent they will meet.
- Since this is within the Zambian infrastructure it may not have generalizability outside of this setting.

3. Innovation:

Strengths

- Using existing infrastructure and collaborations that were used for cervical cancer screening intervention.
- The use of discrete choice experiments is an innovative method of intervention design for youth-friendly services.
- Development of age based developmental interventions for 5 different age groups

Weaknesses

4. Approach:

Strengths

- The SHIELD intervention is theory based and will be developed with the use of community and youth advisory boards based on existing evidence based interventions Stepping Stones and Families Matter!
- Weekly meeting with peer navigators to monitor fidelity of the intervention.
- The use of voice-enabled components and tablets with headphones to reduce stigma and assist with younger adolescents who may not be as literate.
- Given the complexity of the interventions a cluster randomized trial is appropriate.
- The use of propensity scoring to adjust for baseline differences in the cluster sample is a strength.
- The primary endpoints of retention in care and viral suppression are a strength.
- The investigators will be conducting a cost-effective analysis using previously validated models.

Weaknesses

- It is not clear how the discrete choice experiment attributes and levels were chosen. Although the attributes seem important the levels are less clear. When tailoring youth-friendly services there are potential other factors that may influence adolescent engagement.
- Enrolling 1800 individuals in 12 months while completing Aims UG3 – 2 and UG3 – 3 to meet the UG3 milestone seems overly ambitious. It would be strengthened if the investigators documented prior success in such enrollment.

- There is no preliminary data presented making it difficult to determine the feasibility of such a large and rapid enrollment.

5. Environment:

Strengths

- Zambian Ministry of Health, RTI International, The Population Council, and the University of North Carolina have sufficient resources to conduct this proposal and have collaborated together previously and provide strong letters of support.
- The research team has more than a decade of experience working in Zambia with the target population.

Weaknesses

— Responses for Protections for Minor Participants, Human Subjects, Vertebrate Animals, and
— Biohazards:
— A response for Inclusion of Women, Minorities and Children **is required from reviewers** for applications proposing Human Subjects Research

Protections for Minor Participants:

- Appropriate

Protections for Human Subjects:

- No concerns

Data and Safety Monitoring Plan (Applicable for Clinical Trials Only):

- No concerns

Inclusion of Women, Minorities and Children:

- Sex/Gender:
- Race/Ethnicity:
- For NIH-Defined Phase III trials, Plans for valid design and analysis:
- Inclusion/Exclusion of Children under 18:
- No concerns

Vertebrate Animals:

- Not Applicable

Biohazards:

- Not Applicable

Applications from Foreign Organizations:

Select Agent Research:

Resource Sharing Plans:

Authentication of Key Biological and/or Chemical Resources:

Budget and Period of Support:

Recommended budget modifications or possible overlap identified:

Additional Comments to Applicant (Optional):

THE FOLLOWING SECTIONS WERE PREPARED BY THE SCIENTIFIC REVIEW OFFICER TO SUMMARIZE THE OUTCOME OF DISCUSSIONS OF THE REVIEW COMMITTEE, OR REVIEWERS' WRITTEN CRITIQUES, ON THE FOLLOWING ISSUES:

PROTECTION OF HUMAN SUBJECTS: ACCEPTABLE

INCLUSION OF WOMEN PLAN: ACCEPTABLE

INCLUSION OF MINORITIES PLAN: ACCEPTABLE

INCLUSION OF CHILDREN PLAN: ACCEPTABLE

COMMITTEE BUDGET RECOMMENDATIONS: The budget was recommended as requested.

Footnotes for 1 UG3 HD096908-01; PI Name: SUBRAMANIAN, SUJHA

NIH has modified its policy regarding the receipt of resubmissions (amended applications). See Guide Notice NOT-OD-14-074 at http://grants.nih.gov/grants/guide/notice-files/NOT-OD-14-074.html. The impact/priority score is calculated after discussion of an application by averaging the overall scores (1-9) given by all voting reviewers on the committee and multiplying by 10. The criterion scores are submitted prior to the meeting by the individual reviewers assigned to an application, and are not discussed specifically at the review meeting or calculated into the overall impact score. Some applications also receive a percentile ranking. For details on the review process, see http://grants.nih.gov/grants/peer_review_process.htm#scoring.

MEETING ROSTER

National Institute of Child Health and Human Development Special Emphasis Panel
EUNICE KENNEDY SHRIVER NATIONAL INSTITUTE OF CHILD HEALTH & HUMAN DEVELOPMENT
ZHD1 DSR-N (52)
04/09/2018

Notice of NIH Policy to All Applicants: Meeting rosters are provided for information purposes only. Applicant investigators and institutional officials must not communicate directly with study section members about an application before or after the review. Failure to observe this policy will create a serious breach of integrity in the peer review process, and may lead to actions outlined in NOT-OD-14-073 at https://grants.nih.gov/grants/guide/notice-files/NOT-OD-14-073.html and NOT-OD-15-106 at https://grants.nih.gov/grants/guide/notice-files/NOT-OD-15-106.html, including removal of the application from immediate review.

CHAIRPERSON(S)

BAUMAN, LAURIE J, PHD
PROFESSOR
DEPARTMENT OF PEDIATRICS
ALBERT EINSTEIN COLLEGE OF MEDICINE
BRONX, NY 10461

MEMBERS

DELANY-MORETLWE, SINEAD, MBBS, PHD
DIRECTOR
SCHOOL OF CLINICAL MEDICINE
UNIVERSITY OF WITWATERSRAND, JOHANNESBURG
JOHANNESBURG
SOUTH AFRICA

GUILAMO-RAMOS, VINCENT M, PHD
PROFESSOR
CENTER FOR LATINO
ADOLESCENT & FAMILY AND ADOLESCENT AIDS PROGRAM
MONTEFIORE MED CENTER
NEW YORK UNIVERSITY
NEW YORK, NY 10003

LANE, TIM, PHD
ASSOCIATE PROFESSOR
CENTER FOR AIDS PREVENTION STUDIES
UNIVERSITY OF CALIFORNIA, SAN FRANCISCO
SAN FRANCISCO, CA 94158

LUSENO, WINFRED K, PHD
RESEARCH SCIENTIST
PACIFIC INSTITUTE FOR RESEARCH AND EVALUATION
CHAPEL HILL, NC 27514

MARHEFKA, STEPHANIE LYNN, PHD
ASSOCIATE PROFESSOR
COLLEGE OF PUBLIC HEALTH
UNIVERSITY OF SOUTH FLORIDA
TAMPA, FL 33620

PALUMBO, PAUL E., MD
PROFESSOR
DEPARTMENTS OF PEDIATRICS AND MEDICINE
GEISEL SCHOOL OF MEDICINE
DARTMOUTH COLLEGE
LEBANON, NH 03756

SANDERS, RENATA A, MD
ASSISTANT PROFESSOR
DIV. OF GENERAL PEDIATRICS & ADOLESCENT MEDICINE
JOHNS HOPKINS SCHOOL OF MEDICINE
BALTIMORE, MD 21287

SANTA MARIA, DIANE M, MSN, DRPH
ASSISTANT PROFESSOR
SCHOOL OF NURSING
UNIVERSITY OF TEXAS HEALTH SCIENCE CENTER
HOUSTON, TX 77030

SIMONI, JANE, PHD
PROFESSOR
DEPARTMENT OF PSYCHOLOGY
UNIVERSITY OF WASHINGTON
SEATTLE, WA 98195

VENTUNEAC, ANA MARIA, PHD
RESEARCH SCIENTIST
ASSISTANT PROFESSOR
DEPARTMENT OF MEDICINE – INFECTIOUS DISEASES
ICAHN SCHOOL OF MEDICINE AT MOUNT SINAI
ANNENBERG BUILDING FLOOR 23 ROOM, 1468 MADISON AVE
NEW YORK, NY 10018

WECHSBERG, WENDEE M, PHD
DIRECTOR
SUBSTANCE ABUSE TREATMENT EVALUATIONS
AND INTERVENTIONS
RESEARCH TRIANGLE INSTITUTE INTERNATIONAL
RESEARCH TRIANGLE PARK, NC 27709

WOLFMAN, VANESSA AUDREY, MD, MPH
HENRY M. JACKSON FOUNDATION
TECHNICAL DIRECTOR FOR PEDIATRIC AND PMTCT
CLINICAL SERVICES
6720A ROCKLEDGE DR, SUITE #400
BETHESDA, MD 20817

ZANONI, BRIAN C., MD
INSTRUCTOR
HARVARD MEDICAL SCHOOL
MASSACHUSETTS GENERAL HOSPITAL
55 FRUIT STREET
BOSTON, MA 02114

SCIENTIFIC REVIEW OFFICER

HOUSTON, KIMBERLY LYNETTE, MD
SCIENTIFIC REVIEW OFFICER
SCIENTIFIC REVIEW BRANCH (SRB)
EUNICE KENNEDY SHRIVER NATIONAL INSTITUTE
OF CHILD HEALTH AND HUMAN DEVELOPMENT
BETHESDA, MD 20892

EXTRAMURAL SUPPORT ASSISTANT

EDWARDS, MAURICE L.
EXTRAMURAL SUPPORT ASSISTANT
SCIENTIFIC REVIEW BRANCH, DER
EUNICE KENNEDY SHRIVER NATIONAL INSTITUTE OF
CHILD HEALTH AND HUMAN DEVELOPMENT, NIH, DHHS
BETHESDA, MD 20817

Consultants are required to absent themselves from the room during the review of any application if their presence would constitute or appear to constitute a conflict of interest.
